# Editorial: Knowledge of transposable elements

**DOI:** 10.3389/fcell.2024.1360137

**Published:** 2024-01-11

**Authors:** Yabin Guo

**Affiliations:** Guangdong Provincial Key Laboratory of Malignant Tumor Epigenetics and Gene Regulation, Medical Research Center, Sun Yat-sen Memorial Hospital, Sun Yat-sen University, Guangzhou, China

**Keywords:** transposabe element, transposon, retrotransposon, genome, evolution

Transposable elements (TEs, transposons), also known as jumping genes, are mobile elements in the genomes. Complex eukaryotic genomes often contain a large number of TE sequences. TEs play important roles in the maintenance of genome stability, regulation of gene expression, genome evolution, development, aging, and cancerogenesis. Due to the fact that TEs mostly exist in the form of repetitive sequences with multiple copies, they have posed many challenges to research, resulting in the long-term neglect of their functions. In recent years, the rapid development of high-throughput sequencing technologies has greatly advanced the research on TEs, especially the long-read third-generation sequencing, which has provided great convenience for transposon mapping. This Research Topic explores issues such as *de novo* insertions of retrotransposons in human normal cells, the relationship between TEs and aging, amplification of DNA transposons, and the silencing of TEs in gigantic genomes. Transposable element biology research teams from different countries have presented their latest cutting-edge research in this Research Topic through excellent articles.

Woronzow et al. investigated SVA *de novo* insertions and mitochondrial DNA insertions in senescent human lung fibroblasts, and found that the chromosomal structural variations caused by these insertions, similar to epigenetic reshaping, are markers of cell senescence, suggesting the impact of these variations on cell proliferation during cellular aging.

It is known that the retrotransposition of LINE-1 (L1) is one of the causes of human brain mosaicism, and the expression of L1 protein promotes the retrotransposition of non-autonomous retrotransposons such as SVA, further promoting mosaicism. Möhner et al. used RDA combined with deep sequencing techniques to detect SVA *de novo* insertions in different brain regions, and found that new insertions mainly occur in the frontal and midbrain regions, with insertion sites predominantly located in GC-rich and transposon-enriched regions. This study provides valuable information for the study of human brain development and psychiatric disorders.

DNA transposons use a cut-and-paste transposition mechanism, which theoretically does not involve replication, but the copy number of DNA transposons does change, and the underlying mechanisms are still unclear. Redd et al. used yeast as a model to study the factors influencing the copy number of mPing, derived from rice. Since DNA transposons are widely used as transgene vectors and in gene therapy, studying the changes in their copy numbers is of practical value.

There is an equilibrium between the amplification of TEs and their control by the host. However, in some gigantic genomes, the majority of the composition consists of TEs, which seems paradoxical. Wang et al. studied the piRNA pathway in *Ranodon sibiricus*, an amphibian with a 21 Gb genome, providing new clues to this question.

In conclusion, this Research Topic focuses on the forefront of transposable element research, emphasizing the connection between TEs and important issues such as development, aging, and evolution, in order to reveal the multifaceted roles of transposable elements.

